# Pea-Derived Raffinose-Family Oligosaccharides as a Novel Ingredient to Accelerate Sour Beer Production

**DOI:** 10.1021/acs.jafc.4c06748

**Published:** 2025-02-05

**Authors:** Philipp Garbers, Hans Andreas Brandal, Aksel Vardeberg Skeie, Gard W. Karlsnes, Paula Varela, Catrin Tyl, Bjo̷rge Westereng

**Affiliations:** 1 Faculty of Chemistry, Biotechnology and Food Science, Norwegian University of Life Science, Ås 1433, Norway; 2 Sensory and Consumer Sciences, Norwegian Institute of Food, Fisheries and Aquaculture Research, Ås 1433, Norway

**Keywords:** sour beer, cocultures, pulses, Brettanomyces, Lactobacillus, Lactococcus, sensory

## Abstract

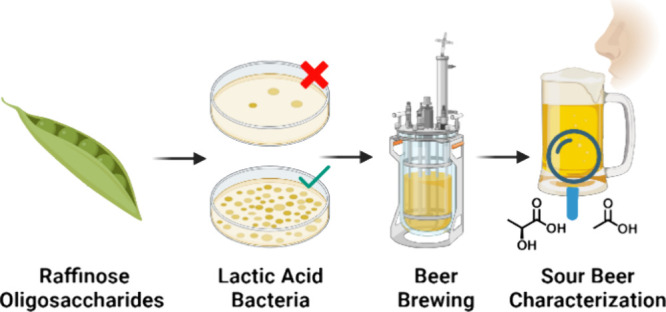

This study investigated raffinose family oligosaccharides (RFOs) derived from pulses as selective carbon sources for sour beer production. Fourteen lactic acid bacteria (LAB) were screened for growth in media supplemented with RFOs. Furthermore, the influence of ethanol and isomerized α-acids on the bacterial growth was investigated. While most LAB grew in the presence of RFOs, few did so in the presence of ethanol and α-acids. Some of the LAB with tolerance to these stressors were then combined with *Brettanomyces claussenii* to create classic-style sour beers with or without RFOs. These were characterized chemically, physically, and sensorially. Sour beers made with RFOs were evaluated as being comparable to a commercial Belgian sour beer for some sensory characteristics. Furthermore, the sensory analysis revealed significantly increased acidity levels and differences in flavor and taste between beers fermented with and without RFOs, which was underpinned by chemical analysis. Crucially, beany off-flavors, which are a common problem with pulse-derived ingredients, did not increase upon RFO addition. Thus, by combining selected LAB with RFOs, we successfully utilized a food sidestream and expanded the possibilities for brewing sour beers in a controlled manner in a short time. This is in contrast to the lengthy process used for traditional sour beers.

## 1 Introduction

Specialty beers, also called craft beers, have become more popular in the last decades, and this has led to an increase in smaller breweries^1^ while the global market for beer also keeps growing.^2^ Among the specialty beers are sour beers, which are characterized by their elevated concentrations of organic acids (mainly lactic acid) and, thus, a sour taste. This is achieved by either adding bacteria (e.g., *Lactobacillus* spp.) and/or certain yeasts (e.g., *Brettanomyces* spp.) alongside common brewing yeasts (*Saccharomyces* spp.),^3^ or by relying on wild fermentations through airborne microorganisms and subsequent long fermentation (>1 year) and maturation in wooden barrels. This is for instance done in brewing of traditional Belgian sour beers (Lambic, Geuze).^3^

However, the brewing industry is facing several challenges, and these may impact sour beer production in numerous ways. Climate change could in the future result in decreasing availability and increasing prices of raw materials for brewing.^4−6^ Moreover, for wild fermented sour beers, the wort is traditionally not cooled by refrigeration but instead left to cool overnight, exposed to the surrounding air through which it comes into contact with wild yeasts and bacteria.^3^ The worldwide increasing ambient temperature^7^ shortens the season with temperatures sufficiently low for the beer to cool down quickly enough and ensure that exposure is limited to desired airborne bacteria and yeast.^8,9^ Hotter temperatures in the summer can furthermore lead to beers that have to be discarded, as has been reported by producers.^9^ Additionally, wild fermentations always carry a certain risk regarding inconsistent quality.^10^

In traditional sour beers, starch from raw wheat serves as a carbon source for specialty yeasts such as *Brettanomyces* spp. during the long fermentation and maturation time. This, however, leads to a complex brewing process because a multistep mashing process or even multiple mashing vessels are needed. Non-sour beers and nontraditional sour beers on the other hand mostly use simpler mashing processes that can be performed in a single vessel.^3^ To reduce the complexity of the process, a previous study from our group explored the possibility of using wood-derived xylo-oligosaccharides as a novel selective carbon source for lactic acid producing bacteria (LAB) in sour beer brewing since these carbohydrates cannot be degraded by common brewer’s yeast (*Saccharomyces cerevisiae*). The produced sour beer had some comparable characteristics to a commercial control.^11^ Thus, use of novel fermentation substrates may allow addressing certain challenges in sour beer production.

**Figure 1:**
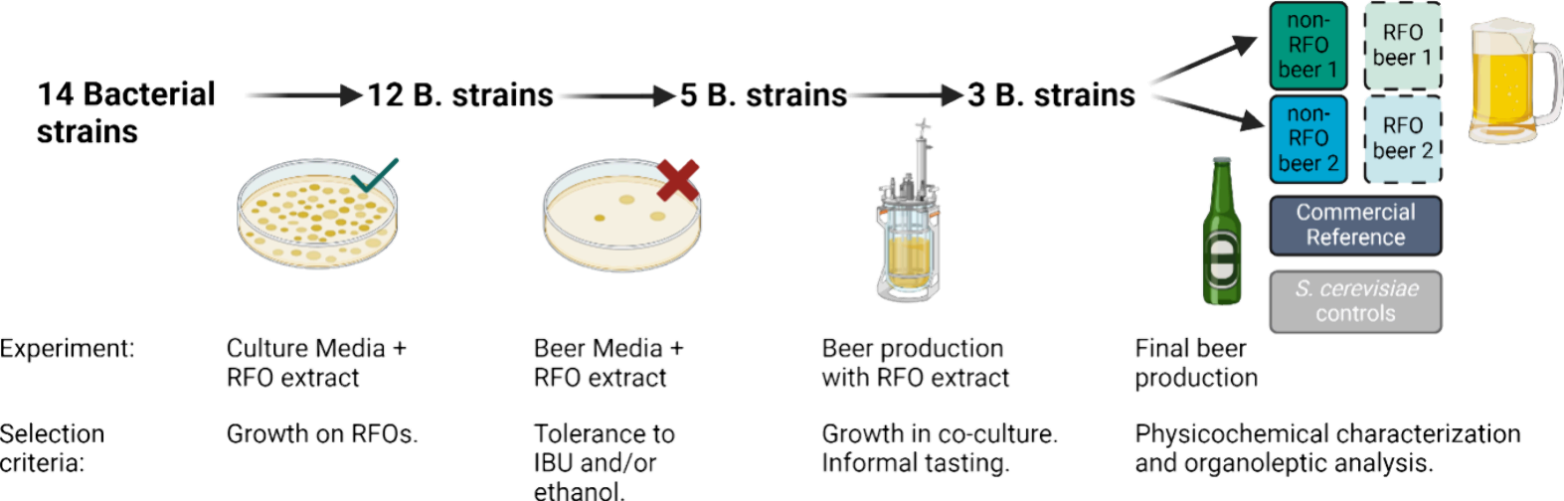
Schematic representation of the study design, showing how 14 bacteria were screened to produce two different batches of RFO-containing beers that were assessed and compared to non-RFO beers and a commercial reference. Created in BioRender. https://BioRender.com/w98x122.

Against this background, this study explored the use of oligosaccharides from pulses in sour beer brewing. Pulses are often mentioned in the same breath as climate change and food security, due to their high protein content, their ability to fixate nitrogen and thus reduce fertilizer use, but also for their nutritional aspects.^12^ However, they also contain so-called raffinose-family oligosaccharides (RFOs) that may pose issues for some consumers. These saccharides are classified as fermentable oligo-, di-, and monosaccharides and polyols (FODMAPs)^13^ and induce gastrointestinal discomfort, especially problematic in connection to irritable bowel syndrome (IBS).^14^ Thus, removal of RFO from pulse-based ingredients (such as pulse proteins) could allow for these products to be consumed by individuals prone to gastrointestinal discomfort. As shown recently, RFOs can easily be extracted from air-classified peas and fava beans.^15^ These pulses are particularly relevant for Scandinavia as they can be grown in colder climates;^16,17^ however, the process can likely also be applied to other pulses such as lentils. Since previous studies have shown the potential of RFOs as a carbon source for LAB,^18−20^ this study screened microorganisms for their ability to grow on media enriched with the obtained RFO extract, with the purpose of identifying strains suitable for beer brewing that are capable of consuming RFOs and tolerant to brewing-related stressors (ethanol and isomerized α-acids). Selected strains were then applied in brewing, and the products were characterized for relevant constituents and beer characteristics. An overview of the study design is shown in Figure 1.

## 2 Material and Methods

### 2.1 Materials

The following strains (all of which have been associated with beer or food in literature) were obtained commercially: *S. cerevisiae* (Fermentis SafAle US-05, Lallemand CBC-1), *Brettanomyces claussenii* (White Laboratories WLP645), *Pediococcus damnosus* (White Laboratories WLP661), *Bifidobacterium animalis* subsp. lactis Bl04, *Lactobacillus delbrueckii* (White Laboratories WLP677), *Lactobacillus rhamnosus* ATCC53103, *Levilactobacillus brevis* BSO 464, and *Lentilactobacillus buchneri* subsp. *silagei CD034*. All other strains were from the collection of Norwegian University of Life Science: The *Lactococcus cremoris* strains were isolated from traditional fermented milk with a mesophilic starter culture, whereas the *Weissella* strains were isolated from soybeans.^21^ The *Lactiplantibacillus pentosus* strains were isolated from olives.^22^

The following analytical grade standards were obtained from Sigma-Aldrich (Merck, Darmstadt, Germany): acetaldehyde, diacetyl, ethyl acetate, 2-butanone, 2-hexanol, 2-methyl-butanal, 2-methyl-1-butanol, 2-methyl-1-propanal, 3-methyl-butanal, 3-methyl-1-butanol, 2-methyl-1-propanol, isobutyl acetate, hexanal, isoamyl acetate, ethyl hexanoate, 3-carene, *R*-(+)-limonene, ethyl heptanoate, ethyl octanoate, β-citronellol, ethyl nonanoate, ethyl decanoate, phenylethyl alcohol, acetoin, acetone, ethanol, 1-butanol, 1-propanol, 2-butanol, dimethyl sulfide, and 2.3-pentadione for analysis described in Section 2.5.3 and maltose, fructose, lactose, glucose, galactose, citric acid, orotic acid, pyruvic acid, succinic acid, lactic acid, formic acid, acetic acid, uric acid, propionic acid, and pyroglutamic acid for analysis described in Section 2.5.2. For RFO analysis which is shown in Section 2.5.1, melibiose (≥98%), d-(+)-raffinose pentahydrate (analytical standard), and verbascose (analytical standard) were from Sigma-Aldrich (Merck, Germany) whereas stachyose hydrate (98%) was obtained from CarboSynth (Biosynth, Staad, St. Gallen, Switzerland).

### 2.2 Oligosaccharide Extraction from Pea Protein Concentrate

The mixture of RFOs used in this study was extracted and purified from pea protein concentrate as described in a recent study.^15^ The extract consisted of 28.6% sucrose, 1% raffinose, 31.4% stachyose, and 16.0% verbascose as well as 19.8% protein (all contents on a dry basis). Briefly, the process consisted of a solubilization step in which the protein concentrate was mixed with water at pH 5. Afterwards, the nonsoluble fraction was removed by a centrifugal separator and the supernatant first filtered over a 0.2–0.4 μm filter and then over a 1 kDa ultrafiltration membrane. The permeate from ultrafiltration was then applied to a nanofiltration membrane, diafiltered, and concentrated, and then the retentate was collected. The collected material was frozen, lyophilized, and stored until further use.^15^

### 2.3 Microbiological Growth Experiments

If not otherwise mentioned, strains of *Lactobacillus* were cultured in De Man–Rose–Sharpe broth (MRS), yeasts were cultured in yeast extract peptone broth (YEP), and strains of *Lactococcus* were cultured in M17 broth, either with 20 g L^–1^ RFO extract as a carbon source or without them. For detailed media composition, see the Supporting Information. Cultures were routinely prepared from frozen stock (−80 °C) in media supplemented with 20 g L^–1^ glucose overnight. LAB were prepared at 37 °C, whereas yeasts were cultured at room temperature (20–24 °C). Cultures were assessed based on their turbidity by measuring the optical density at 600 nm (OD_600_) in a spectrophotometer using media as a reference. Densities larger than 1 were measured by diluting samples and blanks with pure water. For experiments with beer media, beer was produced as described in Section 2.4 but fermented with *S. cerevisiae* and the amount of hops was adjusted to generate the different concentrations of α-acids (IBU) that are specified in Section 3.1.

### 2.4 Beer Production

Wort was produced using a Brewtools B40pro system (Brewtools, Grimstad, Norway) for batches of 20 to 40 L. The malt bill consisted of 2.41 kg (49% w w ^–1^) of each Pilsener malt (barley) and wheat malt from Bestmalz (Heidelberg, Germany). Additionally, rice hulls (2% w w^–1^) were used as filtration aid. The mash was adjusted to pH 5.33 with 80% lactic acid, and two saccharification steps (first at 63 °C, then at 70 °C) were held for 30 min each. Mashing was completed by inactivating enzymes at 78 °C for 10 min. Thereafter, the malt was removed and rinsed with water, 2.14 g of hops (variety: Archer, 1.54% α-acids) were added, and the wort was boiled for 60 min. At the end of the boil, the wort was split in half, and 15 g L^–1^ of RFO extract was added to one-half. Then, the wort was cooled to 20 °C. Batches of 7.5 L of wort were inoculated with equal amounts of the different starter cultures adding up to a total of 150 mL (2% v v^–1^) inoculation volume. After fermentation at 24 °C for 19 days, samples were taken from the beer and the rest was centrifuged. After centrifugation, 7 g L^–1^ of sucrose and 0.1 g L^–1^ of carbonation yeast Lallemand CBC-1 (Montreal, Canada) were added to the supernatant, and the beer was bottled in 0.5 L glass bottles with bottle caps. After 1 week at room temperature, the beers were stored at 4 °C until further evaluation.

### 2.5 Analysis of Beers

#### 2.5.1 Analysis of RFOs with High-Performance Anion-Exchange Chromatography with Pulsed Amperometric Detection (HPAEC-PAD)

A Dionex ICS-6000 System (Thermo Fisher Scientific, Waltham, Massachusetts, USA) consisting of a Dionex ICS-6000 EG, DC, and DP as well as a Dionex AS-AP unit equipped with a Dionex CarboPac PA210-Fast-4 μm column (2 × 150 mm) and a Dionex EGC 500 KOH cartridge was used. Samples (injection volume 0.4 μL) were eluted isocratically (12 mM KOH at a flow of 0.2 mL min^–1^) at a temperature of 30 °C. Chromeleon 7.2.9 software (Thermo Fisher Scientific) was used for data analysis. After comparing different methods (standard addition, external standard curves, matrix-matched calibration), quantification was performed using matrix-matched calibration, meaning that RFO-free beers were used as the solvent for the preparation of calibration curves with known concentrations of the corresponding standards. A selection of fermented beer samples was spiked by adding the same RFO standards and chromatograms compared with starting material to ensure absence of RFO peaks.

#### 2.5.2 High-Performance Liquid Chromatography (HPLC)

Organic acids and saccharides were analyzed based on Dysvik et al.^11^ and Gro̷nnevik et al.^23^ Briefly, beer was filtered (4–7 μm filter paper) and aliquots of 1 mL mixed with 2.5 mL of pure water, 0.2 mL of 0.5 M H_2_SO_4_, and 8 mL of acetonitrile. After vigorous mixing and centrifugation, the supernatant (sample extract) was filtered (0.2 μm syringe filter) before injection. The HPLC system consisted of a 1260 Infinity HPLC (Agilent Technologies, Santa Clara, California, United States) using a refractive index detector for acetic acid and an ultraviolet detector at 220 and 275 nm for all other analytes. The compounds were separated on an Aminex HPX-87H column (Bio-Rad, Hercules, California, United States). The mobile phase consisted of 0.05 M aqueous H_2_SO_4_. Compounds were detected and quantified according to external standard curves, which were run in triplicate and were prepared the same way as samples.

#### 2.5.3 Volatile Analysis via Headspace Gas Chromatography (HSGC)

Volatile compounds were determined via HSGC based on Dysvik et al.^11^ and Gro̷nnevik et al.^23^ Samples were filtered (4–7 μm) to remove CO_2_, and then 10 g was filled into HSGC vials and closed with metal caps with a Teflon-covered septum to preserve the volatile compounds until analysis. Samples were analyzed on a 6890 gas chromatograph with flame ionization detection (Agilent Technologies) equipped with a 7679A autosampler unit (Agilent Technologies) and a CP-SIL 5CB GC column (Varian, Middelburg, Netherlands) with helium as carrier gas. A set of standard compounds was screened based on our previous study of sour beer.^11^ Those that were present in the sour beers prepared in this study were quantified via external standard curves, which were prepared in triplicate. Standards underwent the same sample preparation as for beer samples.

#### 2.5.4 Beverage Analyzer

Chemical parameters of the produced beers were analyzed with an Anton Paar Beverage Analyzer system (Graz, Austria) consisting of the following modules: Density Meter DMA 4500 M, pH 3201, Alcolyzer ME, CarboQC ME, Turbidity Meter HazeQC ME, and a Piercing and Filling Device (PFD). Filtered (<7 μm) samples were filled into pressure-resistant polyethylene terephthalate bottles and were then applied to the system through the PFD module. The instrument-integrated method for beer (Alcolyzer Beer: BEER) was used for all samples.

#### 2.5.5 Titratable Acidity

The titratable acidity was determined based on Beer Method 8 by the American Society of Brewing Chemists.^24^ For the measurement, a known volume (30 mL) of degassed beer was monitored with a pH meter and titrated with 0.1 M NaOH until reaching pH 8.2. Total acidity was expressed in lactic acid equivalents.

#### 2.5.6 Bittering Units

Method B-400.17.110 of the Central European Commission for Brewing Analysis (MEBAK)^25^ was used, for which a 10 mL sample of beer was acidified with 1 mL of 3 M hydrochloric acid, and then the bittering substances were extracted by adding 20 mL of iso-octane and continuous mixing for 15 min. Finally, the mixture was centrifuged, and the nonpolar phase was measured at 275 nm against an iso-octane blank. International bittering units (IBUs) are equal to the absorbance multiplied by 50. One IBU is defined as 1 mg L^–1^ of isomerized α-acids.

#### 2.5.7 Descriptive Analysis

The evaluation of four experimental beers and a commercial sour beer followed ISO 13299:2016. A sensory panel (selected and trained according to ISO 8586:2012(E)) of nine assessors from the Norwegian Institute of Food, Fisheries, and Aquaculture Research conducted a quantitative descriptive analysis (based on Lawless and Heymann^26^). The panel members are hired for the sole purpose of participating as sensory evaluators and are highly trained (on average, 15 years of experience conducting descriptive analyses). A performance check of the panel is conducted at the beginning of each project, based on three qualities: discrimination, repeatability, and agreement. The panel underwent two training sessions, based on which 25 attributes were selected (Supplementary Table 1); the descriptors and definitions were agreed upon, and all assessors were able to discriminate among samples, exhibited repeatability, and reached agreement with other members of the group. Samples were brought from refrigeration to room temperature 1 h before serving, and glasses were rinsed with the respective samples before tasting. Samples were served in a sequential monadic manner, in glasses with three-digit random codes and under red light to minimize visual differences, and were evaluated in two replicates, following a balanced presentation order. The analysis was conducted in a sensory laboratory designed according to ISO 8589:2007(E). For the analysis, EyeQuestion and EyeOpenR (both from Logic8 BV, Utrecht, Netherlands) were used.

### 2.6 Statistical Analysis

Data were analyzed using analysis of variance (ANOVA) followed by Tukey’s honestly significant difference test when significant (*P* < 0.05) differences were detected. Variability among sensory samples was summarized via a principal component analysis (PCA). All statistical analysis was performed in R 4.3.2.

## 3 Results

### 3.1 Screening for Growth

**Figure 2:**
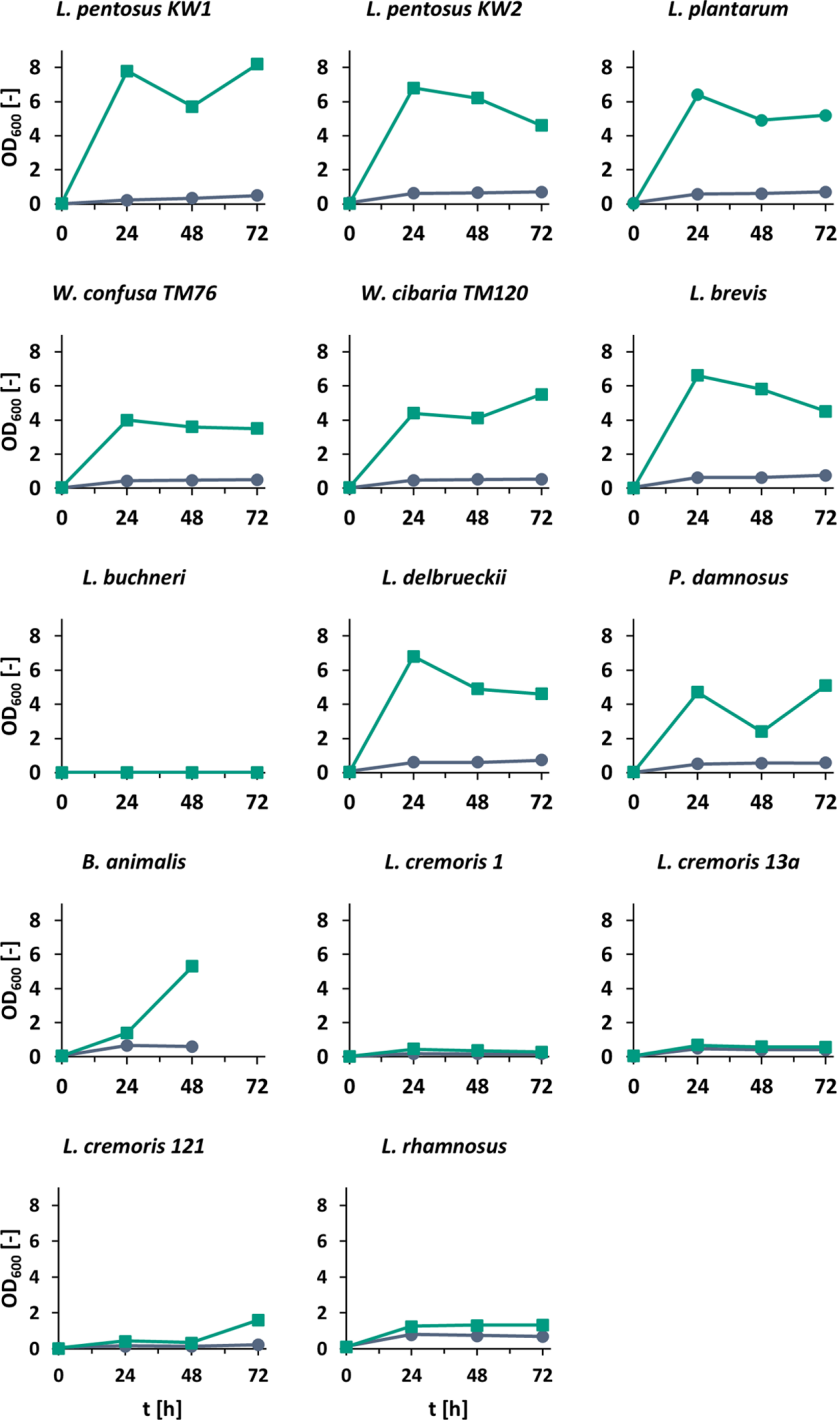
Growth curves of all tested lactic acid bacteria in their respective media without (blue, circle) and with (green, square) 20 g L^–1^ raffinose oligosaccharides at 37 °C. Cultures were grown in duplicates (*n* = 2).

**Figure 3:**
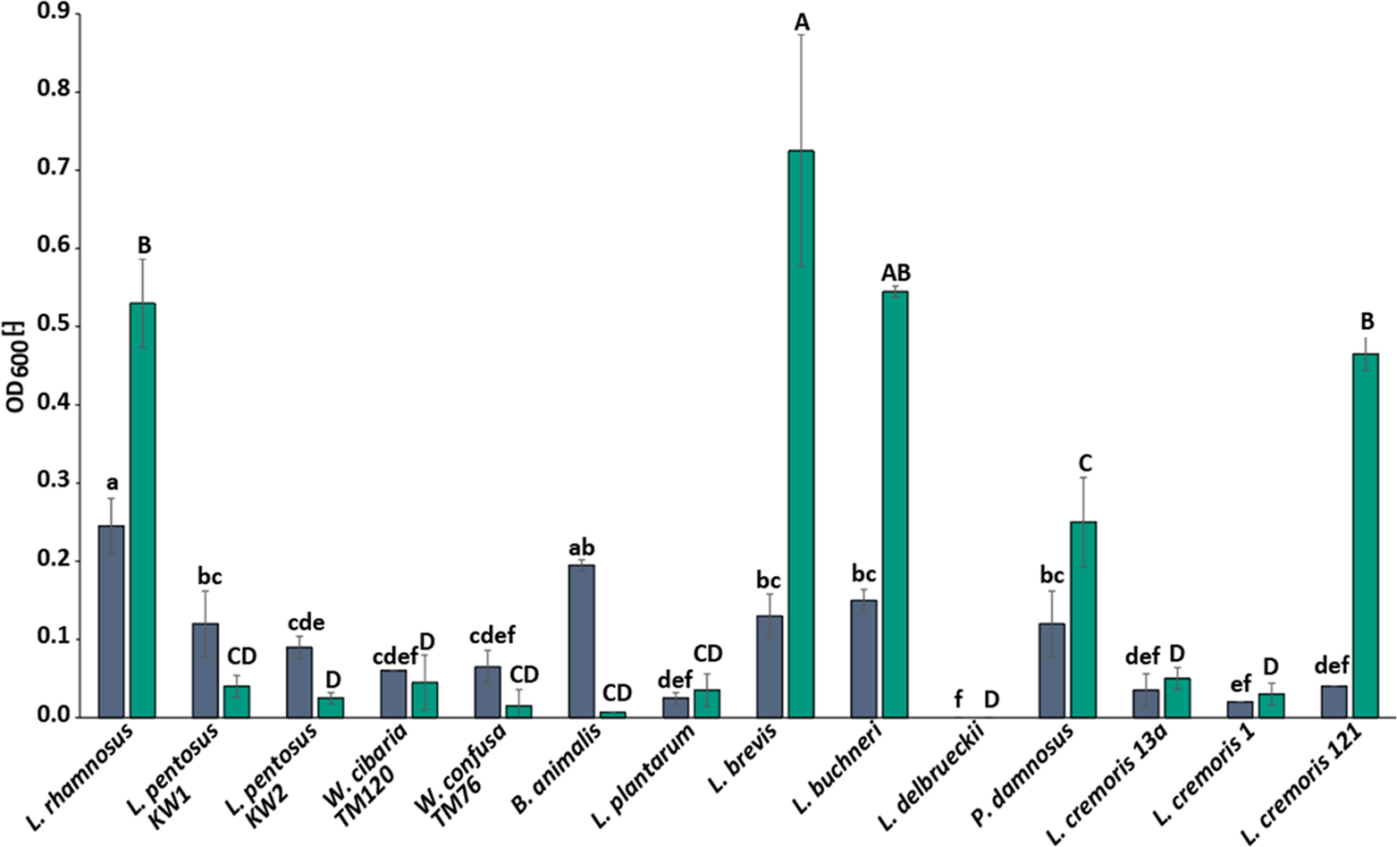
Maximum obtained culture densities during 5 days in beer media (3.27% v v^–1^ alcohol, 6.9 IBU) (blue) and with (green) 15 g L^–1^ raffinose family oligosaccharides (RFOs) (*n* = 2). Different upper- and lowercase letters denote significant differences among samples as determined by Tukey’s honestly significant difference test (*P* < 0.05).

Most LAB strains showed substantial growth in MRS medium over the course of 72 h (Figure 2) when RFOs were supplemented compared with media without a distinct carbon source. *L. pentosus* KW1 reached the highest OD_600_ of 8.20 ± 0.00 whereas *L. rhamnosus* only reached OD_600_ 1.32 ± 0.04. *L. buchneri* did not display any growth, even in the control culture. All other control cultures without a carbon source had similar turbidity, between OD_600_ 0.5 and 0.79. Among the three *Lactococcus* strains cultivated in M17 media, only strain 121 showed a notable increase in growth in the presence of RFOs after 72 h, whereas the difference between control and RFO supplemented media otherwise remained low (Figure 2). Thus, of the 14 strains, 10 showed a positive response to RFOs in the media. However, aside from being capable of using RFOs as substrates, bacteria also needed to tolerate certain stressors to be suitable for beer production. Thus, the growth of 14 strains was also assessed in beer containing 3.27% v v^-1^ alcohol and 6.9 IBU as medium, either with or without 15 g L^-1^ RFOs (Figure 3).

**Figure 4:**
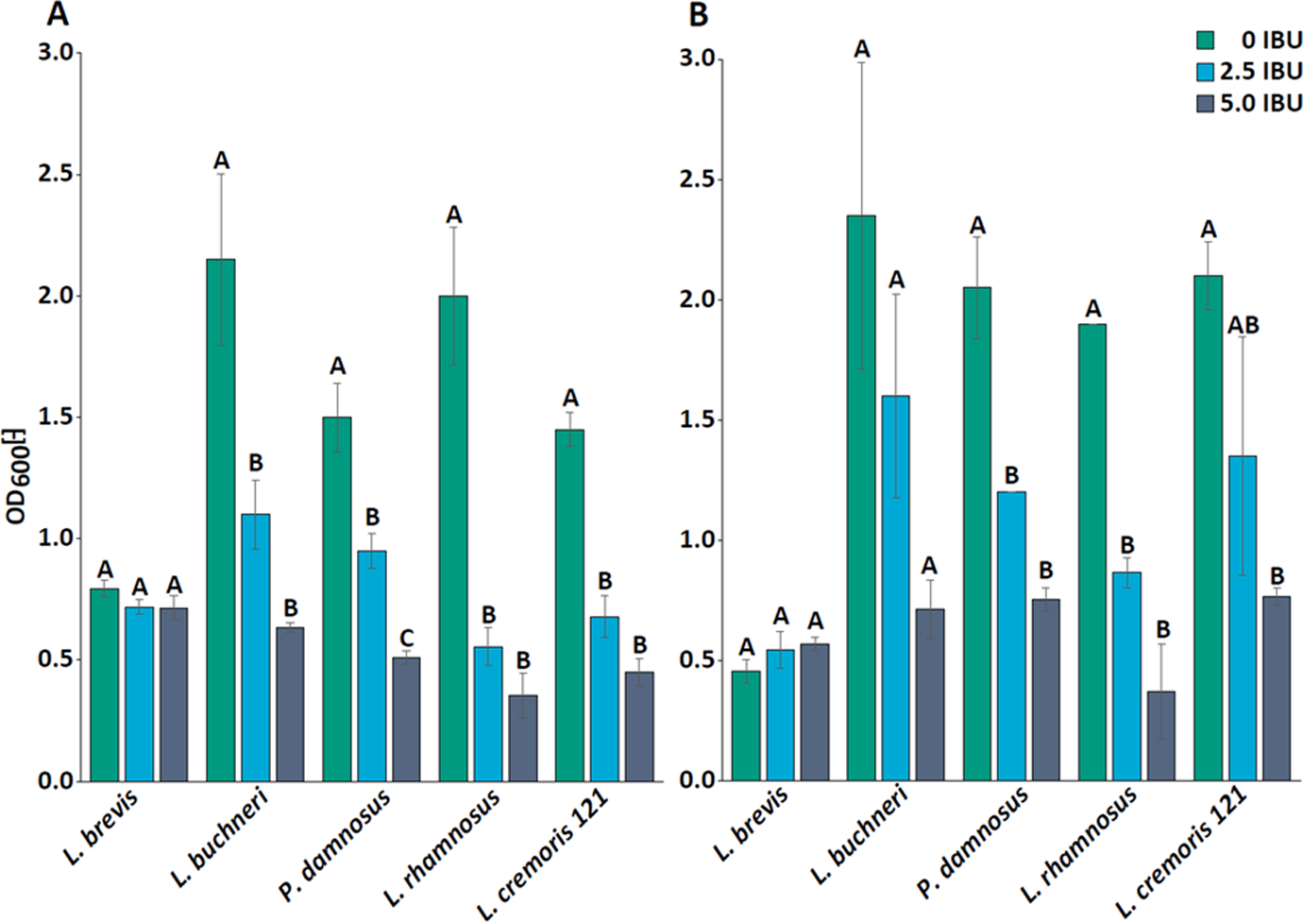
Optical culture density after 7 days in beer with (A) 2.25% v v^–1^ or (B) 4.5% v v^–1^ alcohol and different amounts of hop α-acids (IBU) (*n* = 2). Letters denote significant differences among each strain, as determined by Tukey’s honestly significant difference test (*P* < 0.05).

Overall, the growth in beer medium was substantially lower than that in the respective cultivation media. For example, maximum OD_600_ for *L. brevis* was <1 (Figure 3) compared to >6 in MRS media (Figure 2). Furthermore, upon RFO addition, the OD_600_ only increased in broths with *L. brevis*, *L. rhamnosus*, *L. buchneri*, and *L. cremoris* 121 where it was significantly (*P* < 0.001) higher than in all other cultures. The OD_600_ of *P. damnosus* in RFO-containing beer medium was not significantly different to that of some other strains that exhibited reduced growth in beer medium with RFOs, such as *L. pentosus*KW1. However, it was still higher compared to that of beer medium without RFOs. Thus, *L. brevis*, *L. rhamnosus*, *L. buchneri*, *L. cremoris* 121, and *P. damnosus* were selected for follow-up experiments in beer media with varying ethanol (2.25 or 4.5%v v^-1^) and isomerized α-acids (0, 2.5, or 5 IBU) levels. Growth in the presence of 2.25% ethanol was significantly lower (*P* < 0.05) with 2.5 or 5 IBU than with0 IBU (Figure 4 A, Table 1), except for *L. brevis* (*P* > 0.2). Growth at 4.5% v v^-1^ ethanol (Figure 4 B, Table 1) was similarly affected by IBU but cannot be directly compared to cultures at 2.25% v v^-1^, as dilutions had to be made to reach the lower ethanol concentration. This resulted in lower amounts of available nutrients in media with 2.25% v v than 4.5% v v^-1^, which could have affected the culture densities (OD_600_). The replicates of *L. buchneri* had a relatively large standard deviation at 4.5% v v^-1^, and thus, no statistical difference in growth due to IBU was found. Overall, IBU seemed to be a bigger stressor than ethanol concentration, but both factors still allowed for growth at all tested levels. Based on the knowledge gained during these experiments (Figures 3 and 4), the following decision was made for further brewing: Ethanol levels up to 4.5% v v^-1^ are acceptable, but IBU should be kept at 2.5 IBU to allow for sufficient bacterial growth (Table 1).

**Table 1:** Culture Density for Five Selected Strains after 7 days in Beer with Different Hop (IBU) and Alcohol Concentrations (%v v^–1^)^a^

strain	2.25% v v^–1^ 0 IBU	2.5 IBU	5 IBU	*P*	4.5% v v^–1^ 0 IBU	2.5 IBU	5 IBU	*P*
*L. brevis*	0.80 ± 0.04 ^A^	0.72 ± 0.03 ^A^	0.72 ± 0.05 ^A^	0.215	0.46 ± 0.05 ^a^	0.55 ± 0.08 ^a^	0.57 ± 0.03 ^a^	0.242
*L. buchneri*	2.15 ± 0.35 ^A^	1.10 ± 0.14 ^B^	0.64 ± 0.02 ^B^	0.014*	2.35 ± 0.64 ^a^	1.60 ± 0.42 ^a^	0.72 ± 0.12 ^a^	0.078
*P. damnosus*	1.50 ± 0.14 ^A^	0.95 ± 0.07 ^B^	0.51 ± 0.03 ^C^	0.004**	2.05 ± 0.21 ^a^	1.20 ± 0.00 ^b^	0.76 ± 0.05 ^b^	0.004**
L. rhamnosus	2.00 ± 0.28 ^A^	0.56 ± 0.08 ^B^	0.36 ± 0.09 ^B^	0.005**	1.90 ± 0.00 ^a^	0.87 ± 0.06 ^b^	0.37 ± 0.20 ^b^	0.002**
*L. lactis* 121	1.45 ± 0.07 ^A^	0.68 ± 0.08 ^B^	0.45 ± 0.06 ^B^	0.002**	2.10 ± 0.14 ^a^	1.35 ± 0.49 ^ab^	0.77 ± 0.04 ^b^	0.047*

a Upper- and lowercase letters denote significantly different groups (*P* < 0.05) according to ANOVA and Tukey’s tests per strain and alcohol concentration. Asterisks: *P* = 0.05 < * < 0.01 < ** < 0.001 < ***.

### 3.2 Product Development and Physicochemical Characteristics

During informal tastings of trial brews, the *P. damnosus* strain was excluded due to a strong perceived “buttery” flavor coinciding with the detection of diacetyl (0.372 ppm) in HSGC, which is far higher than the reported taste threshold (0.15 ppm) and mostly undesired in sour beers.^27−29^ Furthermore, the *P. damnosus* beer did not reach pH < 3.8 (data not shown), which is a criterion for sour beers.^3^*L. rhamnosus* was not able to acidify the beer (pH > 3.8) and did not produce any lactic acid (data not shown) during a preliminary cofermentation with yeast and was therefore also excluded.

As the goal was to achieve a sour beer comparable to Lambic or Geuze-type products, *Brettanomyces* spp. was chosen as a yeast strain over *Saccharomyces* spp. due to its known acid tolerance and capability of producing acetic acid, which can be found in these traditional sour beers.^3^ Based on these factors, two batches of wort were produced with the same recipe and divided up: sour beers from batch 1 were fermented with *Brettanomyces claussenii*and *L. cremoris* 121, sour beers from batch 2 with *B. claussenii*, *L. brevis,* and *L. buchneri*. A combination of two bacterial strains was chosen for the second beer, as *L. brevis* was already used as a single strain in the previous study by Dysvik et al.,^11^ and we wanted to avoid repetition as well as achieve higher complexity in aroma. Batch 1 and batch 2 were prepared with and without RFO addition, termed non-RFO beers and RFO beers, respectively. Additionally, batch 1 and 2 were also fermented with *S. cerevisiae* instead of *B. claussenii*. This was done to compare ethanol content and pH, and beers are referred to as *S. cerevisiae* controls. A commercial sour beer was also analyzed as a reference, referred to as commercial reference.

**Figure 5:**
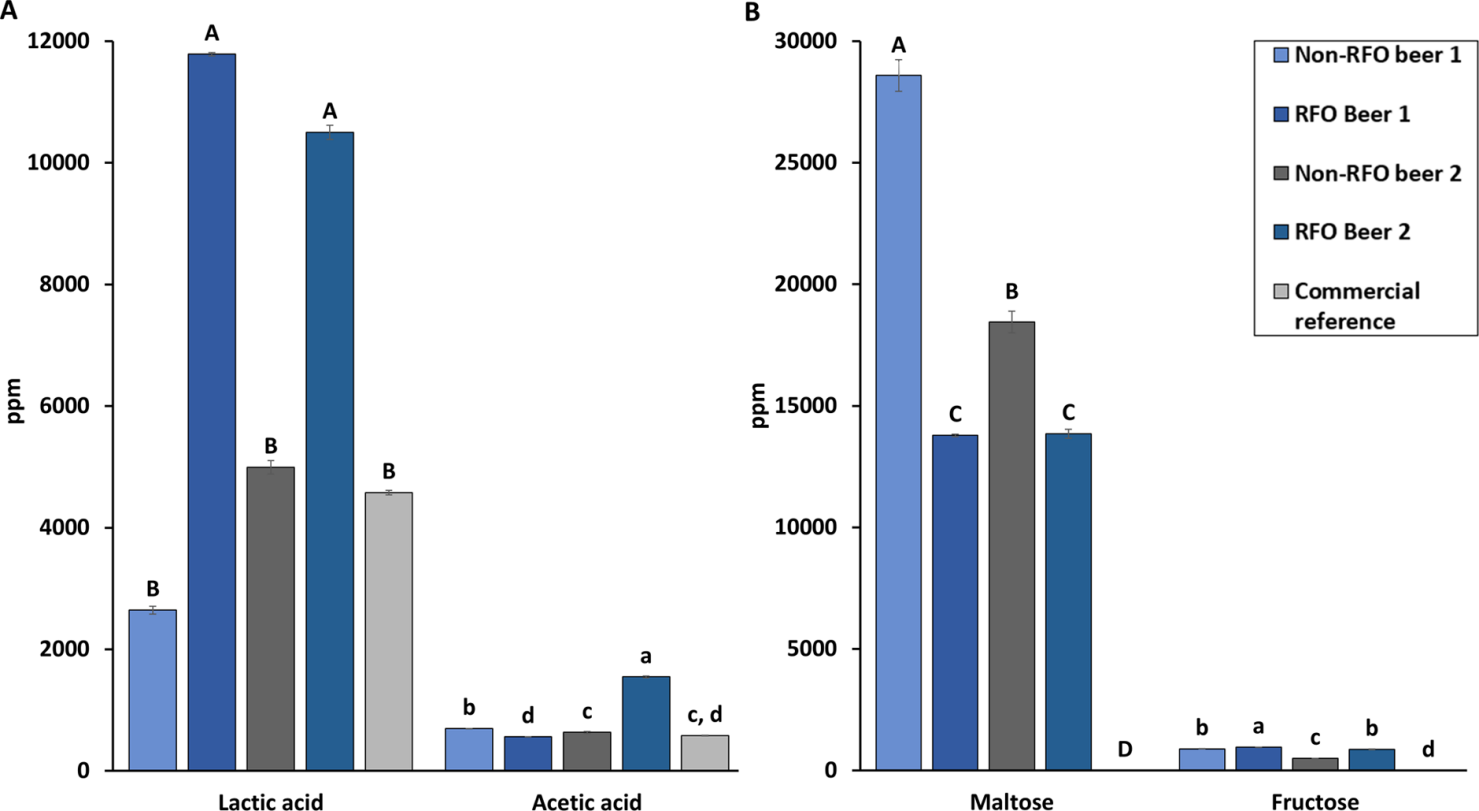
Content of organic acids (A) and residual sugars (B) in the evaluated sour beers (*n* = 2). Letters denote significant differences among samples as determined by Tukey’s honestly significant difference test (*P* < 0.05).

All produced sour beers reached a pH of around 3.5, close to the commercial reference of pH 3.4. However, RFO-sour beers reached notable higher titratable acidities than the commercial reference and non-RFO beers (Table 2). This is in line with RFO beers exhibiting significantly (*P* < 0.05) higher lactic acid contents compared to the commercial reference as well as the non-RFO beers (see Figure 5A). Acetic acid concentrations were however similar, apart from RFO-sour beer 2 containing three times as much as the commercial reference. *S. cerevisiae* controls generally had a higher pH, lower titratable acidity, and organic acid concentrations than their sour-beer counterparts (Table 2). *S. cerevisiae* controls had higher ethanol content (4.04–4.72% v v^-1^) than all sour beers made with *B. claussenii* (1.63–2.41% v v^-1^); however, RFO beers contained more ethanol than non-RFO beers, in line with their higher titratable acidity (Table 2). The low ethanol content of all beers made with *B. claussenii* was also reflected in their residual maltose levels being >10,000 ppm (Figure 5B), whereas no maltose was detected in *S. cerevisiae* controls (data not shown). The RFO beers had significantly lower (*P* < 0.05) maltose levels compared to the non-RFO-beers (−52% RFO beer 1, −25% RFO beer 2). Based on the high concentrations of residual sugars and the low amount of ethanol, the apparent degree of fermentation (ADF) was also low for all *B. claussenii* beers (<40%). Non-sour beers reached above 75–82%, and the highest attenuation (sugar to ethanol conversion) could be found in the commercial control with 84%. However, in contrast to maltose, neither glucose nor RFOs could be detected in any of the sour beers after 19 days of fermentation (Figure S1) and before the sensory analysis, indicating that these carbohydrates were completely consumed by the microorganisms.

**Table 2:** Physical and Chemical Parameters of the Analyzed Beers (ADF = Apparent Degree of Fermentation, EBC = Color According to European Brewery Convention, IBU = International Bitterness Units)

sample	alcohol [% v v^–1^]	density [g cm^–3^]	gravity	original gravity	specific gravity	ADF [% w w^–1^]	CO_2_ [% v v^–1^]	color [EBC]	pH	sugar [°Brix]	bitterness [IBU]	total acidity [mmol L^–1^]
commercial reference	6.46	1.0068	8.48	57.86	1.018	84.64	0.350	19.28	3.40	2.21	16.98	74.3
*S. cerevisiae* control 1	4.72	1.0059	7.30	43.89	1.014	82.69	0.859	5.54	3.93	1.97		39.3
non-RFO beer 1	1.63	1.0284	30.10	42.90	1.033	28.58	0.377	5.64	3.60	7.62	2.75	56.7
RFO beer 1	2.41	1.0268	28.54	47.39	1.032	38.28	0.461	16.10	3.47	7.24	1.90	151.7
*S. cerevisiae* control 2	4.04	1.0060	7.53	38.93	1.013	79.94	0.752	5.42	3.96	2.02		33.3
non-RFO beer 2	1.84	1.0218	23.55	37.98	1.026	36.74	0.381	7.86	3.44	6.00	3.00	73.0
RFO beer 2	2.16	1.0243	26.01	42.91	1.029	38.01	0.354	7.75	3.55	6.61	3.05	125.0

The analysis of volatile compounds (Table 3) revealed that the only compound present in all sour beers above its reported perception threshold (0.030 ppm)^30^ was dimethyl sulfide (DMS); however, levels were similar among samples (Table 3). Acetaldehyde, imparting notes of green apple, was only above its threshold (10 ppm)^28^ in RFO beer 1. Ethyl acetate, which contributes fruity flavor, was above threshold (30 ppm)^28^ in the commercial reference, non-RFO-beer 1, and RFO beer 1 (and close to the threshold in RFO beer 2). Levels of both acetaldehyde and ethyl acetate were higher in RFO beers than in non-RFO beers. The only higher alcohol present above threshold was 3-methyl-1-butanol in the commercial reference and *S. cerevisiae* control 1. The higher alcohols (1-propanol, 2-methyl-1-propanol, 3-methyl-1-butanol, 2-methyl-1-butanol, and 2-hexanol) were mostly below threshold but elevated in beers with higher ethanol content (>4% v v^-1^) compared to the sour beers with less ethanol (<2.5% v v^-1^). 3- and 2-Methyl-butanal were only detected in the commercial sample and the latter also in RFO sour beer 2, but none of them above threshold.^29,31^ 2-Methyl-propanal was only detected in the commercial reference (0.105 ppm), which is slightly above threshold (0.086 ppm^31^). Diacetyl and 2-butanone were not detected in any of the beers.

**Table 3:** Concentration (ppm) of Volatile Compounds in Beer Samples Measured by Head Space Gas Chromatography^a^

sample	acetaldehyde (10–25)^28,29^	dimethyl sulfide^b^ (30)^30^	1-propanol (800)^28,29^	2-butanol (16)^29^	ethyl acetate (30)^28,29^	2-methyl-1-propanol (200)^28^	3-methyl-1-butanol (70)^28^	2-methyl-1-butanol (65)^29^	isobutyl acetate^b^ (1600)^29^	hexanal^b^ (35)^29^	2-hexanol (4)^29^	isoamyl acetate^b^ (1200)^28,29^
commercial reference	6.189 ± 0.657	**37 ± 4**	17.214 ± 0.347	n.d.	**46.601 ± 0.492**	14.532 ± 0.282	**86.973 ± 2.211**	12.045 ± 0.316	32 ± 6	20 ± 5	1.663 ± 0.026	231 ± 16
*S. cerevisiae* control 1	1.031 ± 0.080	**59 ± 4**	27.352 ± 0.728	n.d.	15.838 ± 0.138	91.289 ± 1.162	**70.293 ± 1.213**	19.766 ± 0.453	116 ± 1	n.d.	1.688 ± 0.017	846 ± 25
non-RFO beer 1	1.662 ± 0.081	**58 ± 5**	5.225 ± 0.037	0.323 ± 0.33	**30.633 ± 0.073**	4.791 ± 0.112	17.221 ± 0.097	4.890 ± 0.097	7 ± 2	n.d.	0.236 ± 0.005	125 ± 1
RFO beer 1	**10.060 ± 0.448**	**52 ± 2**	6.676 ± 0.269	0.282 ± 0.000	**42.755 ± 1.223**	4.475 ± 0.196	25.987 ± 1.267	4.667 ± 0.186	6 ± 1	2 ± 0	0.309 ± 0.019	116 ± 6
*S. cerevisiae* control 2	2.975 ± 0.020	**44 ± 4**	23.129 ± 0.069	n.d.	12.469 ± 0.047	76.727 ± 0.305	57.337 ± 0.444	16.996 ± 0.166	94 ± 2	2 ± 0	1.287 ± 0.012	604 ± 6
non-RFO beer 2	2.679 ± 0.080	**58 ± 0**	5.286 ± 0.136	0.280 ± 0.037	15.782 ± 0.243	4.337 ± 0.103	26.871 ± 0.173	4.790 ± 0.041	9 ± 1	3 ± 0	0.244 ± 0.017	219 ± 7
RFO beer 2	5.226 ± 0.265	**42 ± 6**	4.672 ± 0.134	0.344 ± 0.000	27.175 ± 0.381	5.515 ± 0.088	21.124 ± 0.195	4.473 ± 0.005	10 ± 1	2 ± 0	0.186 ± 0.005	102 ± 1

a Values in bold font were above thresholds for human perception (stated in parentheses following the compound).

b Concentration in ppb.

### 3.4 Sensory Evaluation

**Figure 6:**
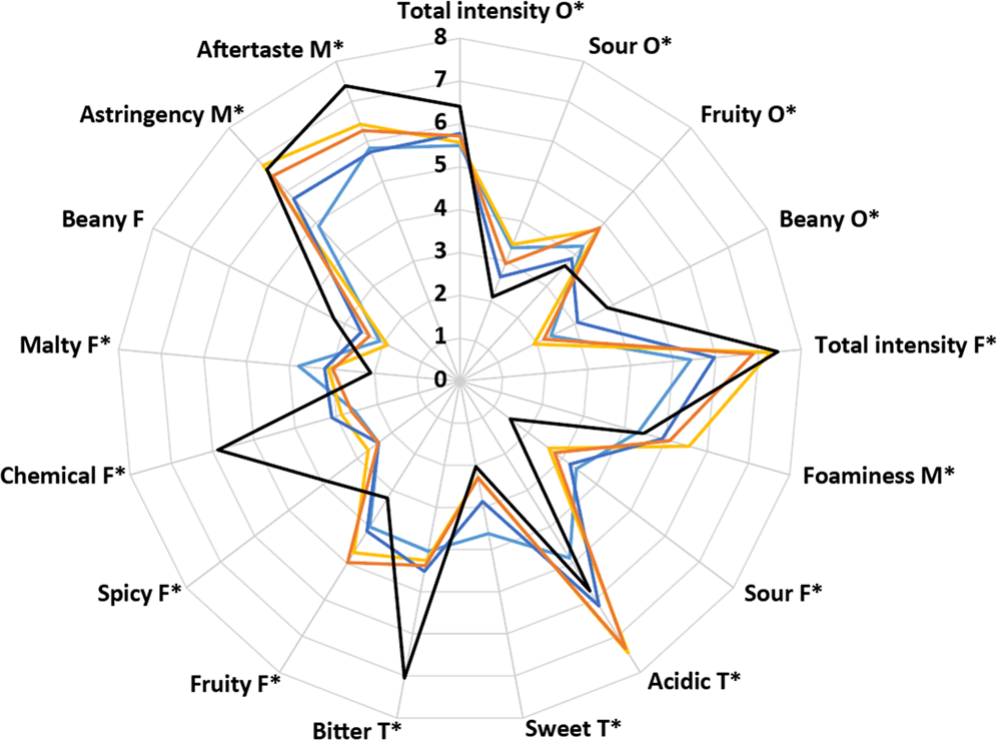
Spider plot of all attributes for which a trained sensory panel perceived significant differences (*P* < 0.05) [*] among the evaluated beers. Beany flavor scores are also shown but were not significantly different. Samples: non-RFO-beer 1 [light blue], non-RFO-beer 2 [dark blue], RFO beer 1 [yellow], RFO beer 2 [orange], and commercial reference [black].

**Figure 7:**
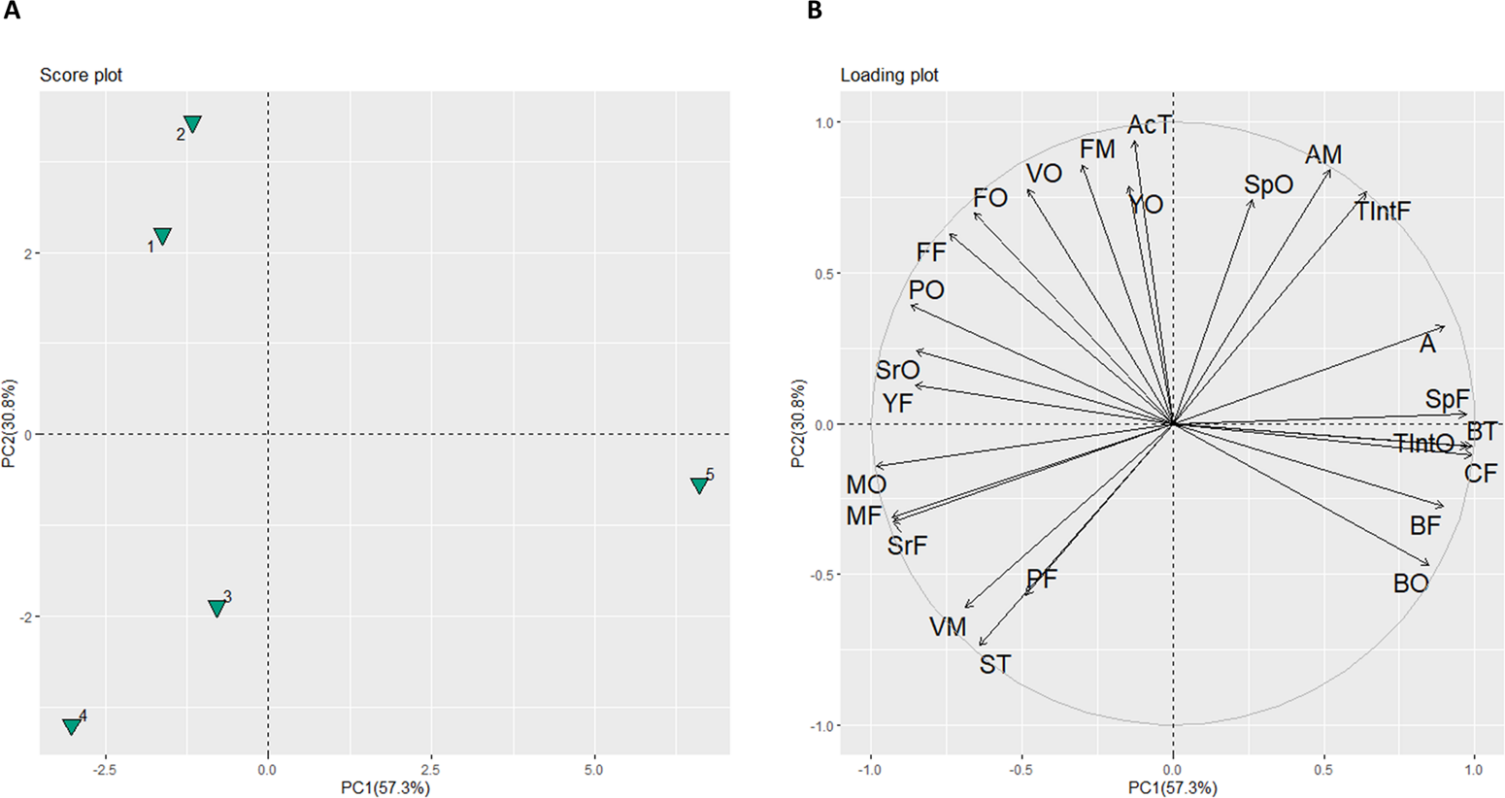
Plots of principal component (PC) analysis summarizing the sensory profiles of the beers. (A) Score plot from principal component analysis (identifiers: 1 = RFO beer 1; 2 = RFO beer 2; 3 = non-RFO-beer 2; 4 = non-RFO-beer 1; 5 = commercial reference) and (B) loading plot – (abbreviations: A = aftertaste; AcT = acidic taste; AM = astringent mouthfeel; BF = beany flavor; BO = beany odor; BT = bitter taste; CF = chemical flavor; FF = fruity flavor; FM = foamy mouthfeel; FO = fruity odor; MF = malty flavor; MO = malty odor; PF = perfumy flavor; PO = perfumy odor; SrF = sour flavor; SrO = sour odor; SpF = spicy flavor; SpO = spicy odor; ST = sweet taste; TIntF = total intensity of flavor; TIntO = total odor intensity; VM = viscous mouthfeel; VO = vinegar odor; YO = yeasty odor; YF = yeasty flavor).

The sensory panel evaluated 25 attributes (Table S1) and found significant differences among beers for 16 of them. The results for these 16 attributes are visualized in Figure 6. The commercial reference exhibited a somewhat distinct pattern, with significantly higher ratings for bitterness, aftertaste, and spicy and chemical flavor as well as significantly lower scores for sour flavor than all other beers (Figure 6). RFO beers were characterized by significantly higher fruity flavor and odor but slightly lower total taste intensities than the commercial reference. RFO beers were evaluated as having significantly higher total taste intensity than non-RFO-beers, and as having significantly higher acidic taste than non-RFO-beers and the commercial reference. The variability among samples in all attributes is presented in Figure 7. Principal component 1 (PC1) accounted for 57.4%, PC2 for 30.7% of the variability. The score plot (Figure 7 A) shows that PC1 separated the commercial reference from other beers, while PC2 differentiated among RFO beers (upper-right quadrant) and non-RFO-beers (lower-right quadrant). Figure 7 B shows that the attributes mostly responsible for this differentiation are in line with those described in the spider plot (Figure 6), *i*.*e*., beany odor, spicy, and chemical flavor of the commercial reference.

## 4 Discussion

For the complete degradation of RFOs, bacteria need two glycoside hydrolases (GH): an invertase (e.g., GH32) and an α-galactosidase (e.g., GH36). According to the carbohydrate-active enzyme database (CAZy),^32^ at least one GH36 is encoded in the genes of *L. pentosus* KW1 & KW2, *B. animalis* subsp. *lactis*, *L. brevis* , and *L. buchneri* , and these strains should hence be able to degrade RFOs at least partially or completely. For *L. buchneri* and *L. brevis* , a complete degradation was shown in beer (Figure S1). This is further supported by a study using *L. brevis* and *L. plantarum* strains in legume sour doughs (including pea), which observed a 62–81% decrease in raffinose within 24 h.^33^ Some *L. plantarum* strains in the data bank^32^ also have the necessary enzymatic tools, and a study on faba beans showed RFO degradation by *L. plantarum*,^34^ implying that also the here-used commercial *L. plantarum* strain for brewing (not in the Databank) contains a GH36, which is reflected in the observed growth behavior (Figure 2). As the *Weissella* strains were isolated from soybeans^21^ and soy contains RFOs,^35^ the presence of an α-galactosidase is also likely and supported by the observed growth response shown in Figure 2. *Weissella* strains of the same species were also reported to degrade RFOs in faba bean fermentations.^19^ The strains’ origins and genome data explain the widely positive response of bacterial growth in Figure 2. Due to the presence of necessary enzymes in the genome, *L. buchneri* remained in the selection of investigated bacteria for the second stage of growth experiments (Figure 1).

The clear change in growth response (significantly reduced OD_600_) when exposed to the stressors ethanol and isomerized α-acids from hops aligns with literature.^36^*L. brevis* and *L. buchneri* have shown good tolerance to the same stressors, whereas the *L. plantarum* strain was severely affected.^37,38^*L. brevis* has also been explicitly used in some studies for its tolerance to brewing-related stress^37,38^ and was highlighted as capable of spoiling non-sour beers (together with *P. damnosus*) and thus as being able to resist some of the antimicrobial factors in beers.^39^ This is also in line with our observations that this strain could utilize RFOs despite the presence of α-acids and ethanol (Figure 3) but was nevertheless influenced by these stressors (Figure 4). The growth response in beer was generally lower than in cultivation media, which relates to nutrient scarcity in beer environments, as yeasts consume most nutrients during beer fermentation.^40^

Nutrient scarcity did, however, not seem to pose an insurmountable challenge for the selected bacteria as all beers had substantial amounts of organic acids. The higher amount of acetic acid in RFO beer 2 than in all other beers (Figure 5A) likely relates to the heterofermentative nature of *L. brevis* and *L. buchneri*(41) allowing them to produce acetic acid from RFOs. Acetic acid levels in the other beers were comparable and presumably solely produced by *Brettanomyces* as it has been shown to produce acetic acid levels of 1000 ppm (1 g L^–1^) in literature.^42^ Comparing RFO beer 1 to non-RFO beer 1, the strictly homofermentative^43^*L. cremoris* presumably produced lactic acid upon RFO addition whereas acetic acid levels remained the same (Figure 5 A). This could explain why lactic acid levels were higher in RFO beer 1 than in RFO beer 2 (Figure 5A).

The higher acidity and ethanol production in the RFO beers compared to non-RFO beers, accompanied by lower maltose levels in RFO beers, hint at an improvement of the fermentation through RFO addition. We hypothesize that yeast mostly converted maltose to ethanol in the presence of RFOs, as *Brettanomyces* is known to mainly produce ethanol and acetic acid.^44^ However, acetic acid increased only in RFO beer 2, which contained the heterofermentative bacteria *L. brevis* and *L. buchneri*. On the other hand, there were no significant differences in lactic acid concentrations between RFO beer 1 and RFO beer 2 (Figure 5). Moreover, RFOs were not detected in either sample (Figure S1). Taken together, this suggests that all LAB indeed metabolized the RFOs and converted them into organic acids. However, transcriptomics and/or proteomics would be necessary to confirm that the bacteria were expressing the enzymes required for RFO degradation.^32^

Even though lactic and acetic acid levels were comparable to or even exceeding those in the commercial reference (Figure 5A), the ethanol content in the produced sour beers remained low (Table 2) and the concentration of residual maltose remained high (Figure 5B). This might have resulted from the timing of yeast and bacteria addition to start fermentations. When pitching yeast either first or simultaneously with bacteria, a previous study with *S. cerevisiae* and *L. brevis* showed no significant difference in ethanol production and residual sugars were only found to be significantly higher when pitching bacteria 24–72 h before the yeast; ethanol formation was however not clearly affected.^37^*Brettanomyces* yeasts have been shown to exhibit slower growth rates than *S. cerevisiae*.^42^ Thus, even though yeast and bacteria were pitched at the same time in our study, the slow growth of *Brettanomyces* may have led to similar outcomes as pitching bacteria before yeast, which in the study of Ciosek and colleagues’^37^ resulted in increased acidity and increased residual sugars in the product. It does, however, not explain the low ethanol production. The manufacturer states an ethanol tolerance of 8–12% for the used strain,^45^ and low pH and high concentrations of organic acids usually do not pose a problem for *Brettanomyces* strains either, as growth down to pH 3 has been reported and lactic acid concentrations of 12–15 g L^–1^ and more were shown to be tolerated by *Brettanomyces* yeasts.^44^ The RFO beers contained 10–12 g L^–1^ lactic acid (Figure 5 A), and this should, therefore, not pose a challenge to the yeast. Moreover, despite the even lower lactic acid concentrations in non-RFO beers (3–5 g L^–1^), these had also low ethanol concentrations (Table 2) and high maltose levels (Figure 5 B), so the acidity might not be the only factor playing a role in this scenario. The *S. cerevisiae* controls reached high attenuation (ADF, Table 2) in the range of the manufacturers data of 78–82% ADF,^46^ and no maltose was detected anymore, showing that the produced wort was indeed fermentable to this degree. Such high attenuation was also expected for the *Brettanomyces* sour beers, which can be seen in the commercial control (84% ADF), but the producer of this particular yeast does not give any information on the strain’s usual attenuation range. A longer fermentation time, as is common in traditional sour beer production, could potentially improve the attenuation.

As discussed, RFOs were consumed during fermentation (see Figure S1) and had an influence on the fermentation performance. It should, however, be pointed out that the extract also contained nitrogenous compounds. Generally, amino acid availability influences the microbial metabolism.^47^ However, a previous study in our group^47^ used *S. cerevisiae*, *L. brevis*, and *L. plantarum* for sour beer production and found that amino acids were not depleted in any of the samples over 21 days. This underlines that the commonly found content of amino acids in wort is not a limiting factor for fermentations. Therefore, we assume that the effect of additional nitrogenous compounds introduced through the RFO extract was minor in this study. Furthermore, amino acids and proteins can also take part in Maillard reactions during boiling,^48^ which produces aroma compounds. However, the RFO extract was only boiled for a few minutes during brewing (Section 2.4), which limits their potential influence on aroma generation. Finally, the extract most likely only contained traces of free amino acids due to its production method, where RFOs were concentrated between an ultrafiltration (1000 Da) and a nanofiltration (ca. 300 Da) membrane, which excludes proteins and enables the washing out of free amino acids by diafiltration.^15^ Overall, we assume that the presence of RFOs as a carbon source influenced the fermentation much more than the nitrogenous material from the RFO extract.

Apart from ethanol, higher alcohols were also detected in all beers and at increased levels when the ethanol concentration was increased, likely due to the production of higher alcohols in yeast (*Saccharomyces*) being directly related to the metabolism.^28^ The lower levels of higher alcohols in here-produced sour beers compared to the commercial reference and *S. cerevisiae* controls aligned with the lower ethanol content in these beers and were most likely due to an overall lower metabolic activity. The fact that DMS was above threshold in all beers could be due to precursors being present in malt and wort and converted either through heat (boiling) or yeast fermentation.^30^ This may explain why there was no difference in DMS concentrations through the addition of RFOs. Another flavor that is associated with malt is hexanal, which is present especially in unmalted cereals, but also in the finished malt and most beers. It is described to lead to a grassy, beany,^49^ or a hay-like flavor.^50^ Beany flavor is considered a major hurdle to more widespread consumption of pulses.^50^ Due to hexanal’s origin in malt, it was detected in most beers, even those without RFOs. In fact, the highest hexanal concentration was found in the commercial reference (Table 3), which is also reflected in the descriptive analysis (Figures 6 and 7) where the highest values for beany odor and flavor were observed for the commercial reference. Fermentation with LAB has however been suggested as a method to mitigate the influence of hexanal on taste, either by removing it or masking the undesired flavor.^51^ There are two potential factors that could have reduced beany flavor or odor in the RFO beers, *i*.*e*., the low presence of hexanal (≤3 ppb) and masking by elevated lactic acid levels. The influence of the lactic acid levels on taste was also observed as increased acidic taste scores for RFO beers in the descriptive analysis (Figure 6). The increased fruity taste detected in RFO beers compared to their non-RFO counter parts (Figures 6 and 7) can be attributed to the increased levels of acetaldehyde and ethyl acetate (Table 3), which are usually described as reminiscent of green apple or fruity in general.^28^ As these are fermentation byproducts of yeasts,^28^ it supports the hypothesis that the addition of RFOs enhanced not only acid and ethanol production but also flavor generation (reflected by increased total intensity flavor, see Figure 6). The increased acidity that was perceived by the tasting panel was expected based on the increased titratable acidity of RFO beers, as this value is considered to be more related to flavor compared to the pH,^52^ which was similar in all beers despite the differences in acid levels. Overall, the results from the volatile and sensory analyses demonstrated that adding a pea-derived ingredient did not impair the aroma profile of the sour beers. This is noteworthy as the flavor of foods and beverages tends to be negatively affected by addition of pulse ingredients and the effect on flavor tends to limit the inclusion amount.^50^ For example, a recent study compared sourdough bread prepared with unfermented pea flour and sourdough made with pea flour fermented with different LAB (e.g., *L. brevis*), sourdough without pea flour, and controls without sourdough.^53^ All samples containing pea flour, regardless of whether it was fermented or not, received higher scores for pea flavor than bread without pea flour. Most also had higher pea aftertaste scores (which, however, were significantly reduced when comparing unfermented pea flour and pea flour fermented with *L. brevis*). This highlights that fermentations with a microorganism of interest may need to be matched to a particular application. Moreover, sour beer appears to be a promising beverage for use of pea-derived RFOs as the metabolite profile of the evaluated LAB seems to align well with product expectations. The remaining olfactory differences between the produced RFO sour beers and the commercial sample could potentially be reduced in future works by adjusting the recipes to compensate for example for differences in bitterness (more hops), malty taste (different malts), and the amount of acid produced (secondary instead of co-fermentation). Even an increased fermentation time for the *Brettanomyces* yeast could be considered as 19 days is a rather short period compared to traditional sour beer fermentations.

In conclusion, this study demonstrated the rational use of RFOs to promote the selective growth of microbes that impart beneficial characteristics to sour beer. While various LAB were able to grow on a pea-derived RFO extract, mainly beer-associated strains continued growing when exposed to the microbial hurdles found in fermented beer, *i*.*e*., ethanol, isomerized α-acids, and nutrient scarcity. The selected bacteria (*L. brevis*, *L. buchneri*, and *L. cremoris*) combined with a *Brettanomyces* yeast led to sour beers with less ethanol but higher lactic acid levels, acidic and fruity taste than the commercial reference. The addition of RFOs increased acid and ethanol production and improved some sensory attributes compared to those of non-RFO beers, thus benefiting the fermentation performance. The beany flavor of pulse-derived ingredients is often considered a hurdle; thus, it is important that RFO beer was evaluated as less beany than the commercial reference in the quantitative descriptive analysis. This study opens up new avenues for producing sour beer with characteristics of traditional styles but in a more streamlined brewing process. Furthermore, a variety of food-borne and food-grade bacteria were shown to utilize the RFO extract. Such extracts could also be obtained from other pulses and used in a wider range of fermented foods and beverages. This could lead to a shift in how RFOs are viewed, *i*.*e*., mitigate dietary concerns and instead regard them as valuable raw material. Thus, the results of this study indicate that pea-derived RFOs can be exploited in unconventional ways to generate products with acceptable sensory properties. Such applications are needed to expand the use of pulses, contributing to a more sustainable food system.

## Supporting Information Available

List of all sensory parameters; list of calibration levels for HPLC, HSGC, and HPAEC; recipes for microbial growth media; and additional HPAEC figure underlining RFO consumption (PDF)

## Abbreviations

ADF: apparent degree of fermentation
CAZy: Carbohydrate-active Enzyme Database
DMS: dimethyl sulfide
EBC: European Brewers Convention
FODMAP: fermentable oligo-, di-, and monosaccharides and polyols
GH: glycoside hydrolases
IBS: irritable bowel syndrome
IBU: International Bitterness Unit
LAB: lactic acid bacteria
MEBAK: Central European Commission for Brewing Analysis
MRS: deMan, Rose, Sharpe media
PFD: piercing and filling device
RFO: raffinose-family oligosaccharides
YEP: yeast extract peptone media

## References

[ref1] Depenbusch L.; Ehrich M.; Pfizenmaier U. Craft beer in Germany. New entries in a challenging beer market. In Economic Perspectives on Craft Beer: A Revolution in the Global Beer Industry, Garavaglia C., Swinnen J., Eds.; Springer International Publishing, 2018; pp 183–210.

[ref2] Swinnen J. B., Devin;. Beeronomics - How Beer Explains the World; Oxford University Press, 2017.

[ref3] Tonsmeire M. American Sour Beers: Innovative Techniques for Mixed Fermentations; Brewers Publications, 2014.

[ref4] Wheeler T.; von Braun J. Climate change impacts on global food security. Science 2013, 341 (6145), 508–513. 10.1126/science.1239402. 23908229

[ref5] Wales A. Making sustainable beer. Nature Climate Change 2014, 4 (5), 316–318. 10.1038/nclimate2220.

[ref6] Xie W.; Xiong W.; Pan J.; Ali T.; Cui Q.; Guan D.; Meng J.; Mueller N. D.; Lin E.; Davis S. J. Decreases in global beer supply due to extreme drought and heat. Nature Plants 2018, 4 (11), 964–973. 10.1038/s41477-018-0263-1. 30323183

[ref7] Lee H.; Calvin K.; Dasgupta D.; Krinner G.; Mukherji A.; Thorne P. W.; Trisos C.; Romero J.; Aldunce P.; Barrett K.; Climate Change 2023: Synthesis Report; IPCC, 2023. 10.59327/ipcc/ar6-9789291691647.

[ref8] Bolton D. Legendary Belgian brewerey Cantillion faces problems as climate change ruins traditional brewing methods. Independent 2015, 05, (11), 2015 https://www.independent.co.uk/climate-change/news/legendary-belgian-brewery-cantillon-faces-problems-as-climate-change-interferes-with-their-traditional-methods-a6722986.html (accessed August 05, 2023)

[ref9] Walsh E. The heat is on // Climate change is coming for Brasserie Cantillion. In Brussels Beer City, www.beercity.brussels, 2018. https://www.beercity.brussels/home/2018/climate-change-brasserie-cantillon-lambic (accessed 05-07-2023)

[ref10] Spitaels F.; Van Kerrebroeck S.; Wieme A. D.; Snauwaert I.; Aerts M.; Van Landschoot A.; De Vuyst L.; Vandamme P. Microbiota and metabolites of aged bottled gueuze beers converge to the same composition. Food Microbiology 2015, 47, 1–11. 10.1016/j.fm.2014.10.004. 25583332

[ref11] Dysvik A.; La Rosa S. L.; Buffetto F.; Liland K. H.; Myhrer K. S.; Rukke E.-O.; Wicklund T.; Westereng B. Secondary lactic acid bacteria fermentation with wood-derived xylooligosaccharides as a tool to expedite sour beer production. J. Agric. Food Chem. 2020, 68 (1), 301–314. 10.1021/acs.jafc.9b05459. 31820631

[ref12] Semba R. D.; Ramsing R.; Rahman N.; Kraemer K.; Bloem M. W. Legumes as a sustainable source of protein in human diets. Global Food Security 2021, 28, 100520 10.1016/j.gfs.2021.100520.

[ref13] Gibson P. R.; Shepherd S. J. Personal view: food for thought--western lifestyle and susceptibility to Crohn’s disease. The FODMAP hypothesis. Alimentary Pharmacology and Therapeutics 2005, 21 (12), 1399–1409. 10.1111/j.1365-2036.2005.02506.x. 15948806

[ref14] Elango D.; Rajendran K.; Van der Laan L.; Sebastiar S.; Raigne J.; Thaiparambil N. A.; El Haddad N.; Raja B.; Wang W.; Ferela A.; et al. raffinose family oligosaccharides: Friend or foe for human and plant health?. Frontiers in Plant Science 2022, 13, 829118 10.3389/fpls.2022.829118. 35251100 PMC8891438

[ref15] Garbers P.; Gaber S. M.; Tyl C.; Sahlstrom S.; Knutsen S. H.; Westereng B. A Pilot-Scale Process for the Extraction of raffinose-Oligosaccharides from Pulse Protein Concentrates. biorxiv.org 2024, 10.1101/2024.04.19.590199.

[ref16] Lizarazo C. I.; Lampi A. M.; Liu J.; Sontag-Strohm T.; Piironen V.; Stoddard F. L. Nutritive quality and protein production from grain legumes in a boreal climate. Journal of the Science of Food and Agriculture 2015, 95 (10), 2053–2064. 10.1002/jsfa.6920. 25242296

[ref17] Svanes E.; Waalen W.; Uhlen A. K. Environmental impacts of field peas and faba beans grown in Norway and derived products, compared to other food protein sources. Sustainable Production and Consumption 2022, 33, 756–766. 10.1016/j.spc.2022.07.020.

[ref18] Karp S. G.; Igashiyama A. H.; Siqueira P. F.; Carvalho J. C.; Vandenberghe L. P.; Thomaz-Soccol V.; Coral J.; Tholozan J. L.; Pandey A.; Soccol C. R. Application of the biorefinery concept to produce L-lactic acid from the soybean vinasse at laboratory and pilot scale. Bioresour. Technol. 2011, 102 (2), 1765–1772. 10.1016/j.biortech.2010.08.102. 20933391

[ref19] Rizzello C. G.; Coda R.; Wang Y.; Verni M.; Kajala I.; Katina K.; Laitila A. Characterization of indigenous *Pediococcus pentosaceus, Leuconostoc kimchii, Weissella cibaria and Weissella confusa* for faba bean bioprocessing. Int. J. Food Microbiol. 2019, 302, 24–34. 10.1016/j.ijfoodmicro.2018.08.014. 30172442

[ref20] Marinaki E.; Kandylis P.; Dimitrellou D.; Zakynthinos G.; Varzakas T. Probiotic Yogurt Production with *Lactobacillus casei* and Prebiotics. Curr. Res. Nutrition Food Sci. 2016, 4 (Special-Issue-October), 48–53. 10.12944/CRNFSJ.4.Special-Issue-October.07.

[ref21] Ng’ong’ola-Manani T. A.; Wicklund T.; Mwangwela A. M.; Østlie H. M. Identification and Characterization of Lactic Acid Bacteria Involved in Natural and Lactic Acid Bacterial Fermentations of Pastes of Soybeans and Soybean-Maize Blends Using Culture-Dependent Techniques and Denaturing Gradient Gel Electrophoresis. Food Biotechnology 2015, 29 (1), 20–50. 10.1080/08905436.2014.996894.

[ref22] Wiull K.; Hagen L. H.; Roncevic J.; Westereng B.; Boysen P.; Eijsink V. G. H.; Mathiesen G. Antigen surface display in two novel whole genome sequenced food grade strains, *Lactiplantibacillus pentosus* KW1 and KW2. Microbial Cell Factories 2024, 23 (1), 19 10.1186/s12934-024-02296-2. 38212746 PMC10782763

[ref23] Grønnevik H.; Falstad M.; Narvhus J. A. Microbiological and chemical properties of Norwegian kefir during storage. Int. Dairy J. 2011, 21 (9), 601–606. 10.1016/j.idairyj.2011.01.001.

[ref24] ASBC Methods of Analysis, A.-M. Beer Method 8: Total Acidity as Titratable Acidity. American Society of Brewing Chemists, 2023. https://www.asbcnet.org/Methods/BeerMethods/Pages/Beer-8-MasterMethod.aspx (accessed 2023 13–07–2023).

[ref25] MEBAK online. Method B-400.17.110. Bittering Units. Mitteleuropäische Brautechnische Analysenkommission (MEBAK\textregistered) e.V., 2020. https://www.mebak.org/en/methode/b-400-17-110/bittering-units/2761 (accessed 2023 2023–07–13).

[ref26] Lawless H. T.; Heymann H.;. Sensory Evaluation of Food; Springer Science & Business Media 2010. 10.1007/978-1-4419-6488-5.

[ref27] Zgherea G.; Stoian C.; Peretz S. Analytical Aspects Regarding the Flavor Compounds in Beer. Journal of Liquid Chromatography & Related Technologies 2011, 34 (13), 1268–1282. 10.1080/10826076.2011.588070.

[ref28] Olaniran A. O.; Hiralal L.; Mokoena M. P.; Pillay B. Flavour-active volatile compounds in beer: production, regulation and control. Journal of the Institute of Brewing 2017, 123 (1), 13–23. 10.1002/jib.389.

[ref29] Tan Y.; Siebert K. J. Quantitative structure-activity relationship modeling of alcohol, ester, aldehyde, and ketone flavor thresholds in beer from molecular features. J. Agric. Food Chem. 2004, 52 (10), 3057–3064. 10.1021/jf035149j. 15137853

[ref30] Bamforth C. W. Dimethyl sulfide – Significance, origins, and control. Journal of the American Society of Brewing Chemists 2014, 72 (3), 165–168. 10.1094/ASBCJ-2014-0610-01.

[ref31] Saison D.; De Schutter D. P.; Uyttenhove B.; Delvaux F.; Delvaux F. R. Contribution of staling compounds to the aged flavour of lager beer by studying their flavour thresholds. Food Chem. 2009, 114 (4), 1206–1215. 10.1016/j.foodchem.2008.10.078.

[ref32] Drula E.; Garron M. L.; Dogan S.; Lombard V.; Henrissat B.; Terrapon N. The carbohydrate-active enzyme database: functions and literature. Nucleic Acids Res. 2022, 50 (D1), D571–D577. 10.1093/nar/gkab1045. 34850161 PMC8728194

[ref33] De Pasquale I.; Pontonio E.; Gobbetti M.; Rizzello C. G. Nutritional and functional effects of the lactic acid bacteria fermentation on gelatinized legume flours. Int. J. Food Microbiol. 2020, 316, 108426 10.1016/j.ijfoodmicro.2019.108426. 31722270

[ref34] Verni M.; De Mastro G.; De Cillis F.; Gobbetti M.; Rizzello C. G. Lactic acid bacteria fermentation to exploit the nutritional potential of Mediterranean faba bean local biotypes. Food Research International 2019, 125, 108571 10.1016/j.foodres.2019.108571. 31554105

[ref35] Kumar V.; Rani A.; Goyal L.; Dixit A. K.; Manjaya J. G.; Dev J.; Swamy M. Sucrose and raffinose family oligosaccharides (RFOs) in soybean seeds as influenced by genotype and growing location. J. Agric. Food Chem. 2010, 58 (8), 5081–5085. 10.1021/jf903141s. 20353171

[ref36] Sakamoto K.; Konings W. N. Beer spoilage bacteria and hop resistance. Int. J. Food Microbiol. 2003, 89 (2–3), 105–124. 10.1016/S0168-1605(03)00153-3. 14623377

[ref37] Ciosek A.; Rusiecka I.; Poreda A. Sour beer production: impact of pitching sequence of yeast and lactic acid bacteria. Journal of the Institute of Brewing 2020, 126 (1), 53–58. 10.1002/jib.590.

[ref38] Modzelewska A.; Jackowski M.; Trusek A. Optimization of beer mixed fermentation using *Saccharomyces cerevisiae* and *Lactobacillus brevis*. European Food Research and Technology 2023, 249 (12), 3261–3269. 10.1007/s00217-023-04365-z.

[ref39] Suzuki K.; Iijima K.; Sakamoto K.; Sami M.; Yamashita H. A Review of Hop Resistance in Beer Spoilage Lactic Acid Bacteria. Journal of the Institute of Brewing 2006, 112 (2), 173–191. 10.1002/j.2050-0416.2006.tb00247.x.

[ref40] Vriesekoop F.; Krahl M.; Hucker B.; Menz G. 125thAnniversary Review: Bacteria in brewing: The good, the bad and the ugly. Journal of the Institute of Brewing 2012, 118 (4), 335–345. 10.1002/jib.49.

[ref41] Zheng J.; Wittouck S.; Salvetti E.; Franz C.; Harris H. M. B.; Mattarelli P.; O’Toole P. W.; Pot B.; Vandamme P.; Walter J.; et al. A taxonomic note on the genus Lactobacillus: Description of 23 novel genera, emended description of the genus *Lactobacillus* Beijerinck 1901, and union of *Lactobacillaceae* and *Leuconostocaceae*. International Journal of Systematic and Evolutionary Microbiology 2020, 70 (4), 2782–2858. 10.1099/ijsem.0.004107. 32293557

[ref42] Abbott D. A.; Hynes S. H.; Ingledew W. M. Growth rates of *Dekkera/Brettanomyces* yeasts hinder their ability to compete with *Saccharomyces cerevisiae* in batch corn mash fermentations. Appl. Microbiol. Biotechnol. 2005, 66 (6), 641–647. 10.1007/s00253-004-1769-1. 15538553

[ref43] Song A. A.; In L. L. A.; Lim S. H. E.; Rahim R. A. A review on *Lactococcus lactis*: from food to factory. Microbial Cell Factories 2017, 16 (1), 55 10.1186/s12934-017-0669-x. 28376880 PMC5379754

[ref44] Serra Colomer M.; Funch B.; Forster J. The raise of *Brettanomyces* yeast species for beer production. Curr. Opin. Biotechnol. 2019, 56, 30–35. 10.1016/j.copbio.2018.07.009. 30173102

[ref45] Labs W. Brettanomyces claussenii. 2024. https://www.whitelabs.com/yeast-single?id=183&type=YEAST&style_type=5 (accessed 2024 09.01.2024).

[ref46] Fermentis. TDS-EN-SafAleTM-US-05. 2024. https://fermentis.com/en/product/safale-us-05/ (accessed 2024 16.01.2024).

[ref47] Dysvik A.; La Rosa S. L.; Liland K. H.; Myhrer K. S.; Ostlie H. M.; De Rouck G.; Rukke E. O.; Westereng B.; Wicklund T. Co-fermentation involving *Saccharomyces cerevisiae* and *Lactobacillus* species tolerant to brewing-related stress factors for controlled and rapid production of sour beer. Frontiers in Microbiology 2020, 11, 279 10.3389/fmicb.2020.00279. 32153550 PMC7048013

[ref48] Hellwig M.; Beer F.; Witte S.; Henle T. Yeast Metabolites of Glycated Amino Acids in Beer. J. Agric. Food Chem. 2018, 66 (28), 7451–7460. 10.1021/acs.jafc.8b01329. 29746116

[ref49] Wang Y.; Guldiken B.; Tulbek M.; House J. D.; Nickerson M. Impact of alcohol washing on the flavour profiles, functionality and protein quality of air classified pea protein enriched flour. Food Research International 2020, 132, 109085 10.1016/j.foodres.2020.109085. 32331653

[ref50] Roland W. S. U.; Pouvreau L.; Curran J.; van de Velde F.; de Kok P. M. T. Flavor Aspects of Pulse Ingredients. Cereal Chem. 2017, 94 (1), 58–65. 10.1094/CCHEM-06-16-0161-FI.

[ref51] Schindler S.; Zelena K.; Krings U.; Bez J.; Eisner P.; Berger R. G. Improvement of the Aroma of Pea (*Pisum sativum*) Protein Extracts by Lactic Acid Fermentation. Food Biotechnology 2012, 26 (1), 58–74. 10.1080/08905436.2011.645939.

[ref52] Tyl C.; Sadler G. D. pH and Titratable Acidity. In Food Analysis, Food Science Text Series; Springer Science & Business Media 2017; pp 389–406.

[ref53] Drakula S.; Novotni D.; Čukelj Mustač N.; Voučko B.; Krpan M.; Vahčić N.; Hruškar M.; Ćurić D. Volatile profile and sensory properties of gluten-free bread with yellow pea flour and sourdough. European Food Research and Technology 2024, 250 (3), 945–960. 10.1007/s00217-023-04439-y.

