# Supplementary material for: Pea-Derived Raffinose-Family Oligosaccharides as a Novel Ingredient to Accelerate Sour Beer Production
Source: J Agric Food Chem. 2025 Feb 5;73(7):4219–30. doi: 10.1021/acs.jafc.4c06748 (PMC11843718; doi:10.1021/acs.jafc.4c06748)
Supplement: Supplementary file 1 — jf4c06748_si_001.pdf [file jf4c06748_si_001.pdf]

Supporting Information

Pea-derived raffinose-family oligosaccharides as a novel ingredient to accelerate sour beer production.

Philipp Garbers[1], Hans Andreas Brandal[1], Aksel Vardeberg Skeie[1], Gard W. Karlsnes[2], Paula Varela[2], Catrin Tyl[1*], Bjørge Westereng[1*]

*bjorge.westereng@nmbu.no, *catrin.tyl@nmbu.no

[1] Faculty of Chemistry, Biotechnology and Food Science, Norwegian University of Life Science, 1433 Ås, Norway

[2] Sensory and Consumer Sciences, Norwegian Institute of Food, Fisheries and Aquaculture Research, 1433 Ås, Norway

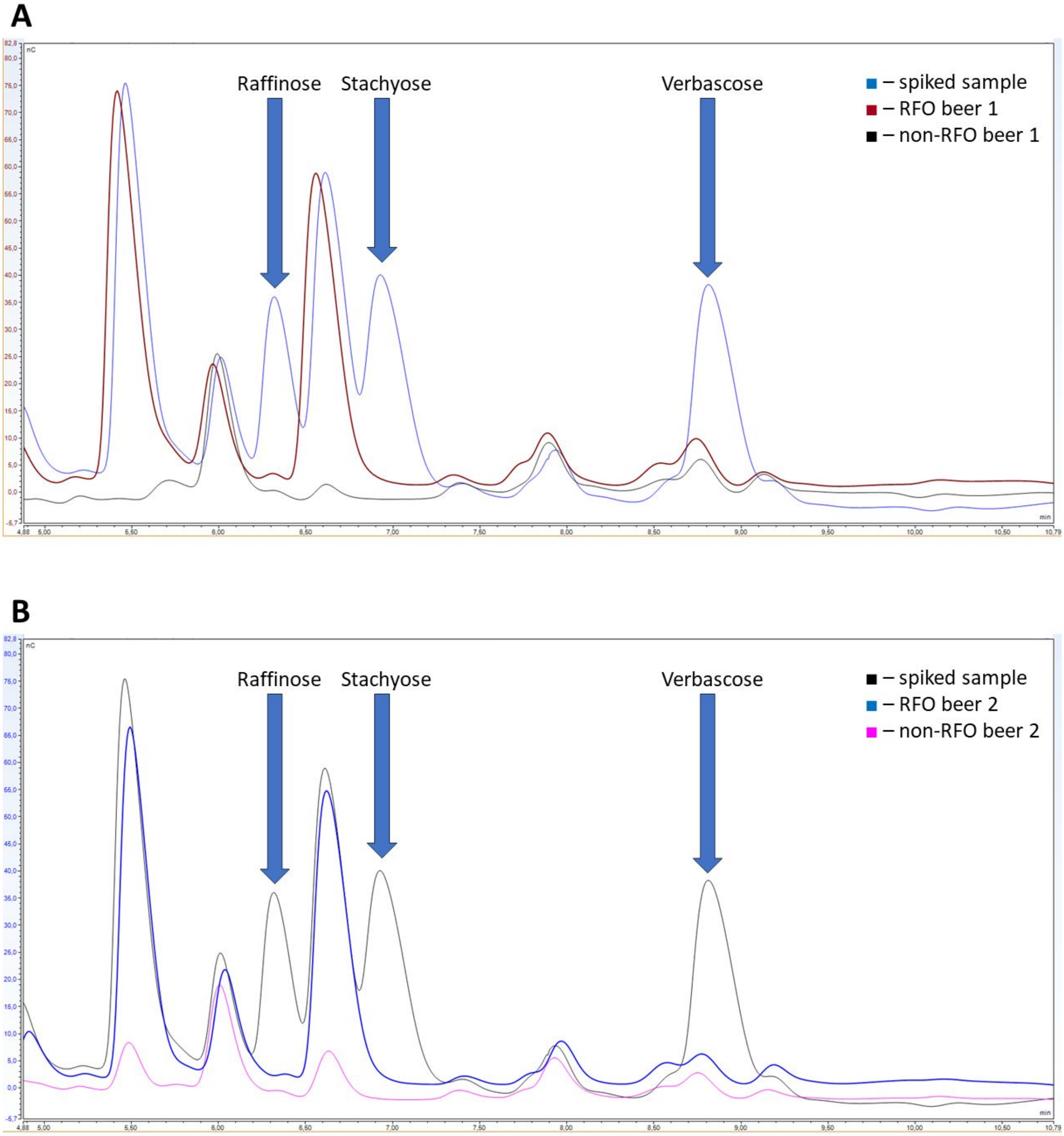

Supplementary Figure 1: Chromatograms from HPAEC analysis of **A** RFO beer 1 and **B** RFO beer 2, compared to their non-RFO counterparts and a sample spiked with standards of raffinose, stachyose and verbascose.

Supplementary Table 1: List of all tested attributes in the sensory analysis including their abbreviations and p-values (significant attributes in bold).

Attribute	Abbreviation	p-value
**Total Intensity Odor**	**TIntO**	**0.006**
**Sour Odor**	**SrO**	**0.006**
Vinegar Odor	VO	0.206
**Fruity Odor**	**FO**	**0.004**
Perfumy Odor	PO	0.097
Yeasty Odor	YO	0.662
Spicy Odor	SpO	0.633
Malty Odor	MO	0.103
**Beany Odor**	**BO**	**0.016**
**Total Intensity Flavor**	**TIntF**	**<0.001**
**Foaminess Mouthfeel**	**FM**	**0.001**
**Sour Flavor**	**SrF**	**<0.001**
**Acidic Taste**	**AcT**	**<0.001**
**Sweet Taste**	**ST**	**<0.001**
**Bitter Taste**	**BT**	**<0.001**
**Fruity Flavor**	**FF**	**<0.001**
Perfumy Flavor	PF	0.536
Yeasty Flavor	YF	0.594
**Spicy Flavor**	**SpF**	**0.001**
**Chemical Flavor**	**CF**	**<0.001**
**Malty Flavor**	**MF**	**<0.001**
Beany Flavor	BF	0.175
Viscosity Mouthfeel	VM	0.158
**Astringency Mouthfeel**	**AM**	**<0.001**
**Aftertaste**	**A**	**<0.001**

Composition of microbial growth media

M-17 Broth without lactose was purchased from Merck (Darmstadt, German) and had the following composition:

0.5 g/L ascorbic acid, 0.25 g/L magnesium sulfate, 5 g/L meat extract, 2.5 g/L meat peptone (peptic), 19 g/L sodium glycerophosphate, 5 g/L soya peptone (papainic), 2.5 g/L yeast extract

DeMan-Rogosa-Sharpe (MRS) broth was prepared from individual microbiology grade components with the following recipe:

2 g/L dipotassium hydrogen phosphate, 0.2 g/L magnesium sulfate monohydrate, 0.05 g/L manganous sulfate tetrahydrate, 8 g/L meat extract, 10 g/L peptone, 5 g/L sodium acetate, 2 g/L triammonium citrate, 4 g/L yeast extract

Yeast Extract Peptone (YEP) broth was prepared in the lab and composed of:

20 g/L peptone, 10 g/L yeast extract (both microbiology grade)

Supplementary Table 2: List of standards and their typical calibration levels (in ppm) for the methods in section 2.5.1, 2.5.2 and 2.5.3

Compound	Instrument	Level 1	Level 2	Level 3	Level 4
Melibiose	HPAEC	5	25	50	125
Raffinose		5	25	50	125
Stachyose		5	25	50	125
Verbascose		5	25	50	125
Maltose	HPLC	9050.525	4525.262	905.052	477.52
Fructose		11518	5759	1151.8	460.72
Lactose		55278	27639	5527.8	2211.12
Glucose		14643	7321.5	1464.3	585.720
Galactose		10622.22	5311.11	1062.222	424.889
Citric acid		2190	1095	219	87.6
Orotic acid		91	45.5	9.1	3.64
Pyruvic acid		155.827	77.914	15.583	6.233
Succinic acid		575	287.5	57.5	23
Lactic acid		9060.251	4530.125	906.025	362.410
Formic acid		462	231	46.2	18.48
Acetic acid		1043	521.5	104.3	41.72
Uric acid		26	13	2.6	1.04
Propionic acid		1097	548.5	109.7	43.88
Pyroglutamic acid		226	113	22.60	9.04
Acetaldehyde	HSGC	20.6	10.3	2.06	
Diacetyl		2.525	1.263	0.253	
Ethylacetate		8.27	4.135	0.827	
2-butanone		8.13	4.065	0.813	
2-hexanol		1.35	0.675	0.135	
2-methyl-butanal		0.756	0.378	0.076	
2-methyl-1-butanol		8.18	4.09	0.818	
2-methyl-1-propanal		1.49	0.745	0.149	
3-methyl-butanal		0.854	0.427	0.085	
3-methyl-1-butanol		38.52	19.26	3.852	
2-methyl-1-propanol		30.95	15.475	3.095	
Isobutyl acetate		0.279	0.14	0.028	
Hexanal		0.288	0.144	0.029	
Isoamyl acetate		1.565	0.783	0.157	
Acetoin		96	48	9.6	
Acetone		4.41	2.205	0.441	
Ethanol		27.29	13.645	2.729	
1-propanol		17.25	8.625	1.725	
2- butanol		5.37	2.685	0.537	
Dimethylsulfide		0.11	0.055	0.011	
2.3-pentadione		3.4	1.7	0.34	
